# Psychometric evaluation of the Japanese version of the fear of pain questionnaire-III and its association with dental anxiety: a cross-sectional study

**DOI:** 10.1186/s12903-023-03273-8

**Published:** 2023-08-12

**Authors:** Mika Ogawa, Teppei Sago, Hirokazu Furukawa, Akihiro Saito

**Affiliations:** 1 Section of Anesthesiology, Department of Diagnostics and General Care, Fukuoka Dental College, Fukuoka, Japan; 2 Department of Dental Anesthesiology, Kyushu Dental University, Kitakyushu, Japan; 3 Department of Clinical Psychology, Naruto University of Education, Tokushima, Japan; 4 Department of Business Administration, The University of Kitakyushu, Kitakyushu, Japan

**Keywords:** Fear of pain, Dental anxiety, Item response theory, Structural equation modeling

## Abstract

**Background:**

Fear of pain is a significant concern related to chronic pain and its impact on daily functioning. It is also associated with dental anxiety, highlighting its relevance in dental practice. This study aimed to validate the Japanese version of the Fear of Pain Questionnaire-III (FPQ-III) and explore its relationship with dental anxiety.

**Methods:**

400 participants completed the Japanese version of the FPQ-III, with 100 participants re-evaluated after one month. Convergent validity was tested against dental anxiety and pain catastrophizing, while discriminant validity was assessed by examining general anxiety and depression correlations. Confirmatory factor analysis was used to examine the factorial validity of the FPQ-III and a shortened version of the FPQ-III (FPQ-9). Item response theory was applied for each subscale to estimate the discriminative power of each item and draw a test information curve. Structural equation modeling (SEM) was used to investigate the relationship between fear of pain and dental anxiety.

**Results:**

Data from 400 participants (200 women, 44.9 ± 14.5 years) were analyzed. The FPQ-III showed good internal validity, intra-examiner reliability, discriminant validity, and convergent validity. Confirmatory factor analysis results supported a three-factor structure, and the FPQ-9 showed a good fit. Test information curves demonstrated that the FPQ-9 maintained high accuracy over a similarly wide range as the FPQ-III. SEM revealed that fear of minor pain was associated with dental anxiety via fear of medical pain even in individuals without painful medical or dental experiences (indirect effect 0.48 [95% CI: 0.32–0.81]). Fear of severe pain tended to be higher in individuals with chronic pain compared to those without (latent mean values 0 vs. 0.27, p = 0.002) and was also associated with dental anxiety via fear of medical pain in women (indirect effect 0.15 [95% CI: 0.01–0.34]).

**Conclusion:**

The Japanese version of the FPQ-9 demonstrated high reliability and validity, making it a valuable tool in dental clinical and research settings. It provides insights into the fear of pain among individuals with chronic pain and dental anxiety, informing potential intervention strategies.

## Introduction

Pain-related fear is a psychological factor that plays a role in developing and maintaining chronic pain [1]. According to the gate-control theory, the central nervous system can influence pain perception [2]. As a biopsychosocial model of pain, chronic pain is considered to develop through a complex interaction of psychological, social, and biological factors [3]. In dentistry, pain-related fears contribute to maintaining oral-facial pain and are associated with dental treatment anxiety [4]. Therefore, assessing fear of pain is crucial in chronic pain research and dental clinical practice.

Self-report measures for pain-related fear or anxiety include the Pain Anxiety Symptom Scale [5] and the Fear of Pain Questionnaire-III (FPQ-III)[6]. The Pain Anxiety Symptom Scale assesses emotional, cognitive, and physiological changes during the pain experience. The FPQ-III is a brief questionnaire that assesses fears of potentially painful experiences of various intensities in daily life.

The FPQ-III is a self-report questionnaire comprising 30 items that assess the intensity of fear of minor pain, severe pain, and medical pain. It has demonstrated high reliability and some construct validity [6]. It has shown high correlations with catastrophic thinking and fear of dental anxiety but low correlations with general anxiety and depression, suggesting that these are distinct constructs [7–9]. There have been reports of a three-factor structure for factorial validity [6], a four-factor structure [10], a six-factor structure [11], and a three-factor structure assuming residual correlation [9]. Two shortened versions have been developed to date [10, 12]. Most studies on the FPQ-III have targeted the general population, with few studies in patients with chronic pain [12, 13].

One limitation of the reliability and validity of conventional methods is that they are entirely dependent on the characteristics of the target sample [12]. For example, results obtained in healthy individuals cannot be directly applied to a patient group. In contrast, item response theory (IRT) allows for the examination of the characteristics of the scale from the examinees’ ability values and the difficulty of the items rather than being dependent on the sample [14]. The FPQ-III has been translated from English into five languages [7–9, 11, 15], but a Japanese version has not yet been produced. Additionally, to our knowledge, there has been no scrutiny of the items using IRT.

The FPQ-III has three subscales [6]. The first is fear related to severe pain at the level of injury to a body part, such as a car accident or fracture. It is reported to be significantly higher in patients with orofacial pain and may be related to chronic pain, such as predicting the frequency and prolonged pain [16]; the second is fear related to minor pain that does not leave physical consequences, such as cutting fingers with hair or soap in the eyes. Fear of minor pain is the only one of the three subscales linked to three loci in genome-wide association studies [17]. The third is pain related to medical care. Fear of painful medical or dental procedures such as injections in the mouth, in the buttocks, or removing a wart with tweezers. Fear of medical pain is strongly associated with the Fear-Avoidance model as well as minor pain, results in increased anxiety [18]. As described above, each of the three subscales is assumed to have a different psychological role.

In dentistry, fear of pain has been reported to be associated with orofacial pain and anxiety about dental treatment [4, 13]; fear of pain and dental anxiety are related but distinct concepts. Previous studies have indicated a genetic background, with candidate gene approaches suggesting that melanocortin-1 receptor mutations mediate the relationship between dental anxiety and fear of pain [4], and genome-wide association studies have shown that fears of minor pain share a partial genetic basis with dental anxiety [17]. Factors contributing to dental anxiety are predominantly fear conditioning due to painful dental treatment experiences [19]. Furthermore, there are individual differences in the susceptibility to develop dental anxiety, and one phenotype of this vulnerability is considered to be the Fear of Pain [19]. However, how the three subscales relate to dental anxiety remains open to investigation. Fears of minor and severe pain are hypothesized to b related factors likely to develop dental anxiety. On the other hand, fear of medical pain is a concept that has much in common with dental anxiety, as the item includes injections into the oral cavity. Therefore, it was hypothesized that fear of medical pain could also be influenced by minor and severe pain and consequently be associated with dental anxiety.

As noted above, painful dental and medical treatment experiences are assumed to influence dental anxiety [20, 21]. In addition, the FPQ-III contains items related to fractures and road accidents and articles related to pain associated with medical treatment, which is hypothesized to be related to the impact of pain-related experiences. Both fear of pain and dental anxiety are reported to be higher among women than men [9]. Dental anxiety is higher among women than men in Japan [22]. Pain anxiety has not yet been measured in Japan, but given that chronic pain patients are more likely to be female in Japan [23], there may be a gender difference. The above suggests that pain-related fears are related to dental anxiety and that pain-related experiences and gender differences confound the relationship. Still, no studies investigating these simultaneously have been found.

The first aim of this study was to develop a Japanese version of the FPQ-III and to examine its reliability and validity in a conventional The second aim was to investigate the relationship between fear of pain and dental anxiety regarding gender and the presence or absence of painful medical or dental experiences.

## Methods

The cross-sectional study was approved by the Ethical Committee of Fukuoka Dental College (approval number: 586).

### Procedure

The original author’s permission was obtained to develop a Japanese-language version. The FPQ-III was translated into Japanese using the back-translation method. Two fluent English speakers, one dentist, and one psychologist, separately translated the FPQ-III into English. Another dentist and a psychologist then merged these translations. An expert fluent in English, experienced in back-translation, retranslated the integrated Japanese version into English. Another translator, who had not been involved in the back-translation process, reviewed the original and back-translated versions.

### Participants

The rule of thumb for multigroup structural equation modeling is 100 cases per group [24]. Therefore, the target sample size was also set at 400. All participants were internet monitors for MSS Inc. (Tokyo, Japan) as convenience sampling. Individuals with severe fear of medical pain and individuals with severe dental anxiety tend to avoid visiting a medical institution [25]. To find out about general trends of fear of pain in Japan, we targeted internet monitors instead of recruiting targeted participants at medical institutions. The overall demographics of the registered monitors were 56% female, 55% over 40 years old, and 54% married; 42% were office workers, 17% part-timers, 10% homemakers and 8% students. Participation criteria were defined as Japanese speakers living in Japan and aged 20 or over. There were no exclusion criteria. To facilitate control for the confounding factors of age and gender, age and gender were equally assigned, and responses were obtained.

A simultaneous email was sent to randomly selected monitors who met the eligibility criteria. When the respondents visited the website for the survey via Email, the policy for using data and protecting personal information was displayed. Informed consent was obtained by pressing the button that stated, ‘I agree to the research,’ which appeared at the end of the explanatory document. Only those who agreed with the procedure were allowed to answer the questionnaire. No missing values are generated due to the system, whereby responses cannot be submitted unless all questions are answered. The survey was closed when 440 participants had completed the questionnaire. MSS Inc. used its own protocols to clean the data and eliminate untruthful respondents. Data for this study was collected over three days in June 2022. For reliability studies using the retest method, the FPQ-III was studied again one month later in 100 randomly selected participants, with a 50% male/female ratio. To reduce non-response bias, we awarded respondents points that could be exchanged for e-money [26]. The survey was conducted anonymously, and the authors did not have access to information that would allow them to identify individuals.

### Measures

All participants were asked to provide details about sociodemographic characteristics (i.e., age, gender, occupation, and educational level) and clinical information (i.e., presence of chronic pain lasting more than three months, Location and frequency of chronic pain). They were also asked whether they had experienced painful dental treatment, painful medical treatment, broken bones, and road traffic accidents. Current pain was measured using the Numerical Rating Scale, which asked respondents to “Please answer the intensity of the pain you have felt most often during the past month, with no pain as 0 and worst pain as 10”.

Fear of pain was measured using the FPQ-III, a 30-item self-administered questionnaire that assesses fear of painful experiences that may be encountered in everyday life and healthcare settings [6]. Each item was obtained on a five-point Likert scale ranging from not at all to extreme, with an overall score ranging from 30 to 150. Three subscales (severe, minor, and medical pain) exist, with ten items each. Good internal consistency, retest reliability, and convergent and discriminant validity are reported. There is a shortened 23-item version by Asmundson et al. [10] and a 9-item version by the original authors [12].

Anxiety and depression were assessed using the Japanese version of the Hospital Anxiety and Depression Scale, a self-administered scale [27]. It consists of seven items on anxiety and seven items on depression and is used for screening purposes in outpatient clinics and health check-ups due to its simplicity. The responses were obtained on a four-point scale. The range is 0–21 points each. The Japanese version has been reported to have high reliability and validity [28].

Dental anxiety was measured with the reliable and valid Japanese version of the Modified Dental Anxiety Scale (MDAS) [29], a 5-item questionnaire that assesses anxiety in five situations: going for treatment tomorrow, sitting in the waiting room, having one’s tooth drilled, having one’s teeth scaled and polished, and receiving a local anesthetic injection. Responses are recorded on a 5-point Likert-type scale ranging from “not anxious” to “extremely anxious” that sum up to a total score (range 5–25). Higher scores indicate greater dental anxiety. The two established factors of MDAS were calculated as anticipatory dental anxiety (items 1 and 2; range = 2–10) and treatment dental anxiety (items 3, 4, and 5; range = 3–15) [30, 31]. The Japanese version of MDAS has been reported to be one factor [32, 33].

Pain Catastrophizing was measured using the Pain Catastrophizing Scale, a valid and reliable Japanese 13-item self-report measure was used to assess three components of catastrophizing: rumination, magnification, and helplessness [34, 35]. Responses were measured with a five-point Likert scale ranging from “strongly disagree” to “strongly agree.” Total scores range from 13 to 65.

### Statistical analyses

All quantitative variables were treated as continuous variables. For the multigroup structural equation modeling described below, those who answered that they had experienced painful medical or dental experiences were defined as the group, namely “NegativeEX”, and the others were defined as the group, namely “Non-Negative EX”. Sample demographics and mean values for the FPQ-III and other scales were calculated stratified by gender. Dental anxiety and pain catastrophizing were assessed using Pearson’s correlation coefficients to investigate convergent validity, and general anxiety and depression to investigate discriminant validity. Reliability was evaluated using internal consistency and retest reliability methods. Cronbach’s α assessed internal consistency of the total scale and subscales; values > 0.70 were considered acceptable [36]. The Intraclass Correlation Coefficient (ICC) was employed to evaluate test-retest reliability. As a rule of thumb, ICC values between 0.61 and 0.80 indicate moderate reliability, and those between 0.81 and 0.90 indicate substantial reliability [37].

#### Item response theory (IRT)

In order to treat the subscales as independent factors in the next structural equation modeling, an item analysis was conducted using item response theory, assuming each of the three subscales was a single factor. Item response theory was utilized to estimate the discriminative power (α) and item difficulty (β) of each item using the Graded Response Model and draw a test information curve [14, 38]. Before the main IRT, categorical factors analysis and a preliminary IRT were conducted to remove items with a factor pattern of less than 0.35, a discriminative power of less than 0.65[39].

#### Structural equation modeling (SEM)

**Fig. 1:**
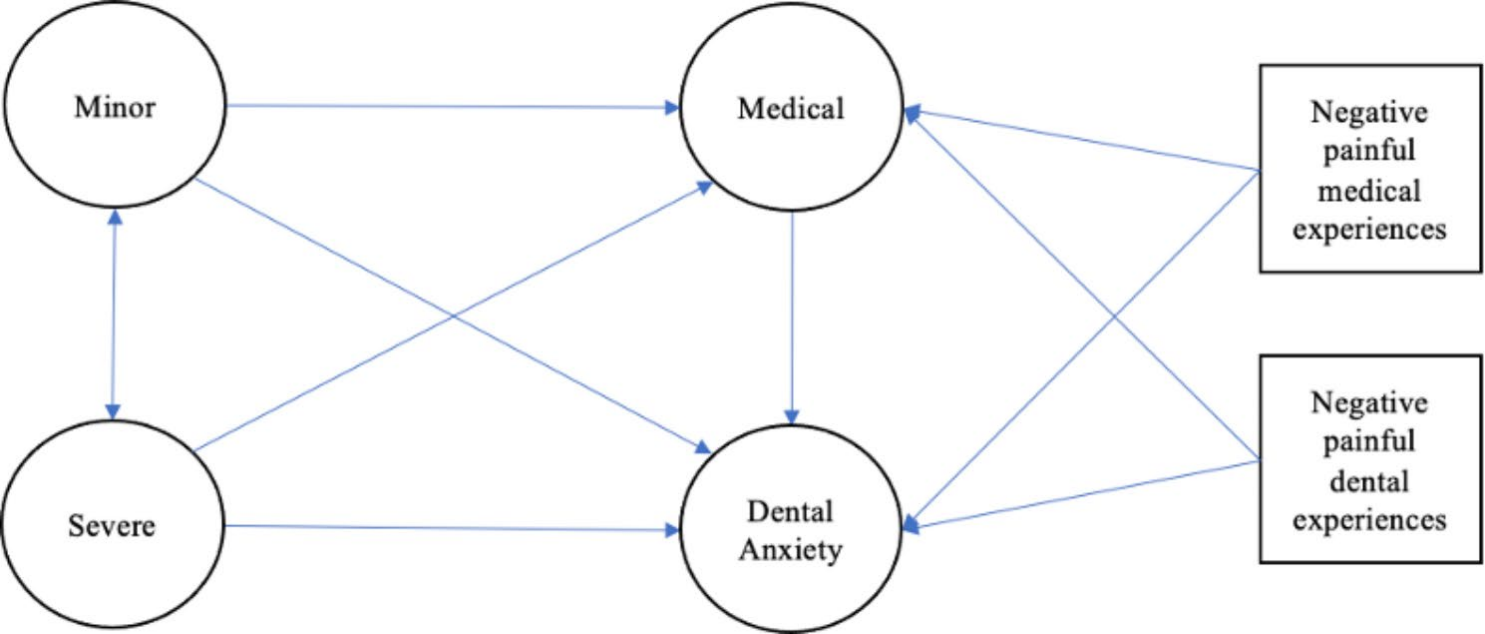
The hypothetical structural equation model for the relationships between dental anxiety, fear of pain, and painful medical or dental experiences

The factorial validity of the FPQ-III and the shortened version was examined using confirmatory factor analysis. SEM was used to assess the relationships among three subscales of FPQ-9, dental anxiety, negative dental experiences, and negative medical experiences. Our hypothetical model is shown in Fig. 1. Prior analyses showed that experience of fracture and traffic accidents did not significantly correlate with fear of pain and dental anxiety, so they were not included in the model. The following fit indices were used: chi-square and its significance, the comparative fit index (CFI), the root mean square error of approximation (RMSEA), and standardized root mean square residual (SRMR). The values of X2/df < 5, CFI > 0.90, and RMSEA < 0.08 indicate a reasonably good fit [24]. The best model has the smallest Akaike information criterion (AIC) and Bayesian information criterion (BIC) [24].

#### Measurement invariance

To examine the equality of the covariance structure across gender, the presence of chronic pain, and the presence of painful medical/dental experiences, we constructed models with different constraints. First, the Configural Invariance Model assumed that the observed variables measuring the nuclear factor are equal across populations. Next, the Metric Invariance Model imposed equality constraints on the factor patterns measuring each factor across populations. The Scalar Invariance Model added further constraints by equalizing the intercepts measuring each factor. Finally, the Strict Invariance Model included equal residuals measuring each item in addition to the constraints in the Scalar Invariance Model.

We compared each model to the previous one based on changes in the Comparative Fit Index (ΔCFI), Root Mean Square Error of Approximation (RMSEA), Akaike’s Information Criterion (AIC), and Bayesian Information Criterion (BIC). We used a cut-off value of 0.010 for ΔCFI to assess the model fit [40].

SPSS Statistics software version 27 (IBM SPSS, Armonk, NY, USA) for description and factor analysis for exploration. IRT and SEM were conducted using R version 4.0.0 (R Foundation for Statistical Computing, Vienna, Austria) and Package “ltm” [41] and “lavaan” [42]. All tests were conducted at a significance level of 0.05.

## Results

A total of 10,000 targets among the 307,722 internet monitors received an email invitation to participate in our survey from the research company. Four hundred forty participants participated in the study, and data from 440 were collected. The survey company’s protocols excluded 40 unserious respondents. Data from 400 subjects who passed the quality checks (200 women [50.0%], mean 44.9 ± 14.5 years) were analyzed. None of the data contained missing data. Socio-demographics of the participants are shown in Table 1. About half of the respondents were company employees, and about half had a university degree. About 35% felt they had chronic pain, which was mild, with a mean score of the Numerical Rating Scale was 2.5.

**Table 1:** Sociodemographic valuables, experiences, the scores of fear of pain and dental anxiety of the participants

		Total (n = 400)	% or S.D.	Male (n = 200)	% or S.D.	Female(n = 200)	% or S.D.
Age		44.9	14.52	45.25	14.91	44.56	14.139
Occupation							
	Salaried employment	208	52.00	138	69.00	70	35.00
	Self-employed	20	5.00	15	7.50	5	2.50
	Housewife	59	14.80	0	0.00	59	29.50
	Part-time worker	51	12.80	7	3.50	44	22.00
	Student	12	3.00	7	3.50	5	2.50
	Unemployed	43	10.80	28	14.00	15	7.50
	Other	7	1.80	5	2.50	2	1.00
Education							
	Junior high Graduate school Other	18	4.50	10	5.00	8	4.00
	High school	123	30.80	58	29.00	65	32.50
	Technical college	57	14.20	18	9.00	39	19.50
	University	192	48.00	109	54.50	83	41.50
	Other	10	2.50	5	2.50	5	2.50
The presence of chronic pain							
	Yes	141	35.30	69	34.50	72	36.00
	I don’t know	22	5.50	9	4.50	13	6.50
	No	237	59.30	122	61.00	115	57.50
Painful dental pain experience							
	No	293	73.30	152	76.00	141	70.50
	I don’t know	19	4.80	10	5.00	9	4.50
	Yes	88	22.00	38	19.00	50	25.00
Painful medical pain experience							
	No	301	75.30	155	77.50	146	73.00
	I don’t know	26	6.50	11	5.50	15	7.50
	Yes	73	18.30	34	17.00	39	19.50
Bone fracture							
	No	263	65.80	122	61.00	141	70.50
	I don’t know	9	2.30	6	3.00	3	1.50
	Yes	128	32.00	72	36.00	56	28.00
Traffic accident							
	No	260	65.00	122	61.00	138	69.00
	I don’t know	12	3.00	6	3.00	6	3.00
	Yes	128	32.00	72	36.00	56	28.00

The translated version of the FPQ-III file is available after contacting the corresponding author.

Mean FPQ-III total scores and subscales were significantly higher for women than men (Table 2). On the other hand, no gender differences were found in the mean values of the MDAS.

**Table 2:** Mean and their gender differences of FPQ-III total scores and the Modified Dental Anxiety Scales

	Total (n = 400)		Male (n = 200)		Female (n = 200)		
	Mean	S.D.	Mean	S.D.	Mean	S.D.	P value
Total	95.66	22.92	91.78	22.67	99.54	22.55	< 0.001
Severe	39.35	7.63	37.44	7.65	41.25	7.13	< 0.001
Minor	26.96	9.07	26.02	8.98	27.89	9.07	0.039
Medical	29.36	9.11	28.32	8.90	30.40	9.22	0.022
MDAS	12.26	5.06	11.92	4.95	12.61	5.16	0.173

Abbreviations: FPQ, Fear of Pain Questionnaire. MDAS, Modified Dental Anxiety Scale

A comparison of the mean FPQ-III with and without chronic pain is shown in Table 3. Fear of severe pain tended to be slightly higher in the group with chronic pain than in the group without chronic pain. However, no differences were observed in fear of minor pain or medical pain.

**Table 3:** A comparison of the mean FPQ-III with and without chronic pain

	With chronic pain (n = 141)		Without chronic pain (n = 259)		
	mean	S.D.	mean	S.D.	P value
FPQ-III	97.02	22.68	94.92	23.06	0.381
Severe	40.61	7.13	38.66	7.82	0.014
Minor	26.79	9.06	27.05	9.09	0.785
Medical	29.62	8.91	29.21	9.23	0.666

Abbreviations: FPQ-III, Fear of Pain Questionnaire

### Reliability and internal consistency

Total scores on the FPQ-III showed good internal validity and within-examiner reliability (Table 4).

**Table 4:** Internal consistency and test-retest reliability

	Cronbach’s α	ICC	ICC (95% CI)
Severe	0.894	0.676	0.555–0.770
Minor	0.918	0.823	0.749–0.878
Medical	0.918	0.854	0.791–0.899
Total	0.958	0.812	0.734–0.870

Abbreviations: ICC, Intraclass Correlation Coefficient

### Validity

Table 5 shows no significant correlation was found between FPQ-III and depression, and a weak correlation with anxiety. These results indicate that the FPQ-III has discriminant validity (r = -0.07, p = 0.16; r = 0.26, p < 0.01, respectively). On the other hand, moderate positive correlations were found between the FPQ-III and dental anxiety and catastrophic thinking, showing convergent validity (r = 0.52, p < 0.01; r = 0.49, p < 0.01, respectively).

**Table 5:** Pearson’s correlation for divergent and convergent validity

	HADS Anxiety	HADS Depression	MDAS	PCS
FPQ total	0.263**	-0.071	0.517**	0.492**
Severe	0.087	-0.260**	0.343**	0.347**
Minor	0.284**	0.016	0.448**	0.426**
Medical	0.306**	0.023	0.568**	0.523**

** p < 0.001

Abbreviations: FPQ, Fear of Pain Questionnaire; HADS, Hospital Anxiety and Depression Scale; MDAS, Modified Dental Anxiety Scale; PCS, Pain Catastrophizing Scale

**Fig. 2:**
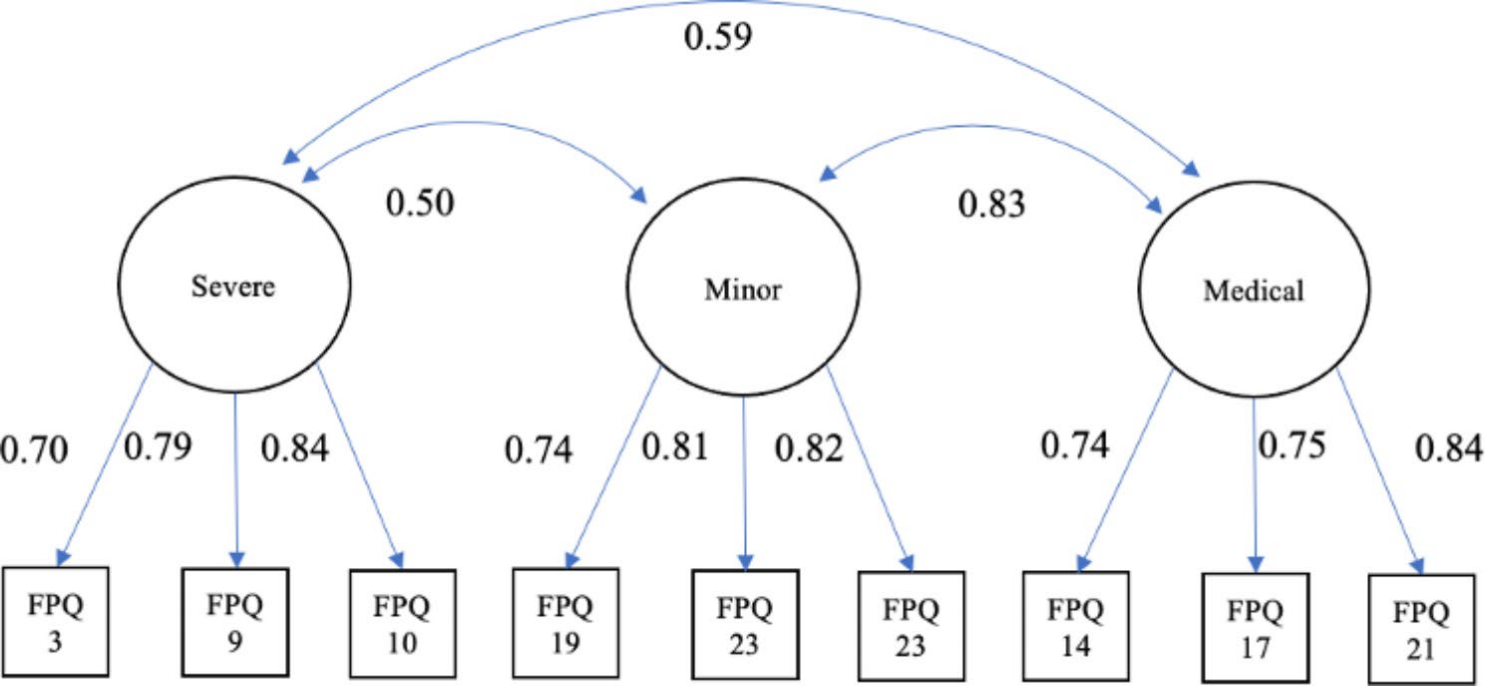
The estimated equation model with standardized coefficients for nine fear of pain questionnaire items FPQ: Fear of Pain Questionnaire

Confirmatory factor analysis showed a poor fit of the three-factor structure (FPQ-J model). The goodness of fit improved when error correlations were set for five items related to injection, fracture, and dentistry, as Di Tella et al. showed [9] (Table 6). However, the CFI remains below the criteria for good conformity; the 23-item shortened version [10] is also not good, despite an improving trend compared to the original. On the other hand, the FPQ-9 [12] showed a good fit (Table 6). Figure 2 shows the results of the SEM for the FPQ-9 model.

**Table 6:** CFA model’s goodness of fit for the Japanese version of FPQ-III

Model	X^2^	df	CFI	RMSEA	SRMR	AIC	BIC
FPQ-J	2096	402	0.80	0.103	0.096	30,429	30,861
FPQ-J with five errors’ covariance	1640	397	0.853	0.088	0.089	29,983	30,434
A 23-item version of FPQ-J	853	164	0.870	0.103	0.083	20,318	20,621
A 9-item version of FPQ-J	63.7	24	0.977	0.064	0.032	9502	9640

Abbreviations: CFI, the comparative fit index; RMSEA, the root mean square error of approximation; SRMR, standardized root mean square residual; AIC, Akaike’s information criterion; BIC, Bayesian information criterion

Table 7 reports the invariance test results with or without chronic pain. A strict model was adopted from the comparison of CFIs. No significant differences in structure, path coefficient, intercept, and error variance were found with and without chronic pain. These results show that latent means can be compared between the two groups. Mean structure values were significantly higher in the group with chronic pain only for fear of severe pain than in the group without chronic pain (0.257, P = 0.002).

**Table 7:** Comparison of FPQ-9 structure in groups with and without chronic pain

Model	X^2^	df	CFI	RMSEA	SRMR	AIC	BIC
Overall model	63.7	24	0.977	0.064	0.029	9521	9640
Configural model	91.1	48	0.975	0.067	0.035	9535	9774
Metric model	99.5	54	0.974	0.065	0.042	9531	9747
Scalar model	108.9	60	0.972	0.064	0.044	9529	9720
Strict model	119.1	69	0.971	0.06	0.044	9521	9676

Abbreviations: CFI, the comparative fit index; RMSEA, the root mean square error of approximation; SRMR, standardized root mean square residual; AIC, Akaike’s information criterion BIC, Bayesian information criterion

### Item analysis

The results of item analysis for each subscale of the FPQ-III showed that all items were within the criterion range for discriminative power, factor patterns and polychoric correlation coefficients. Item difficulty tended to be higher in the Fear of severe pain subscale (Table 8). The shortened 9-item version also showed a similar trend to the FPQ-III, with discriminative power within the criterion range and item difficulty being higher in fear of severe pain (Table 9).

**Fig. 3:**
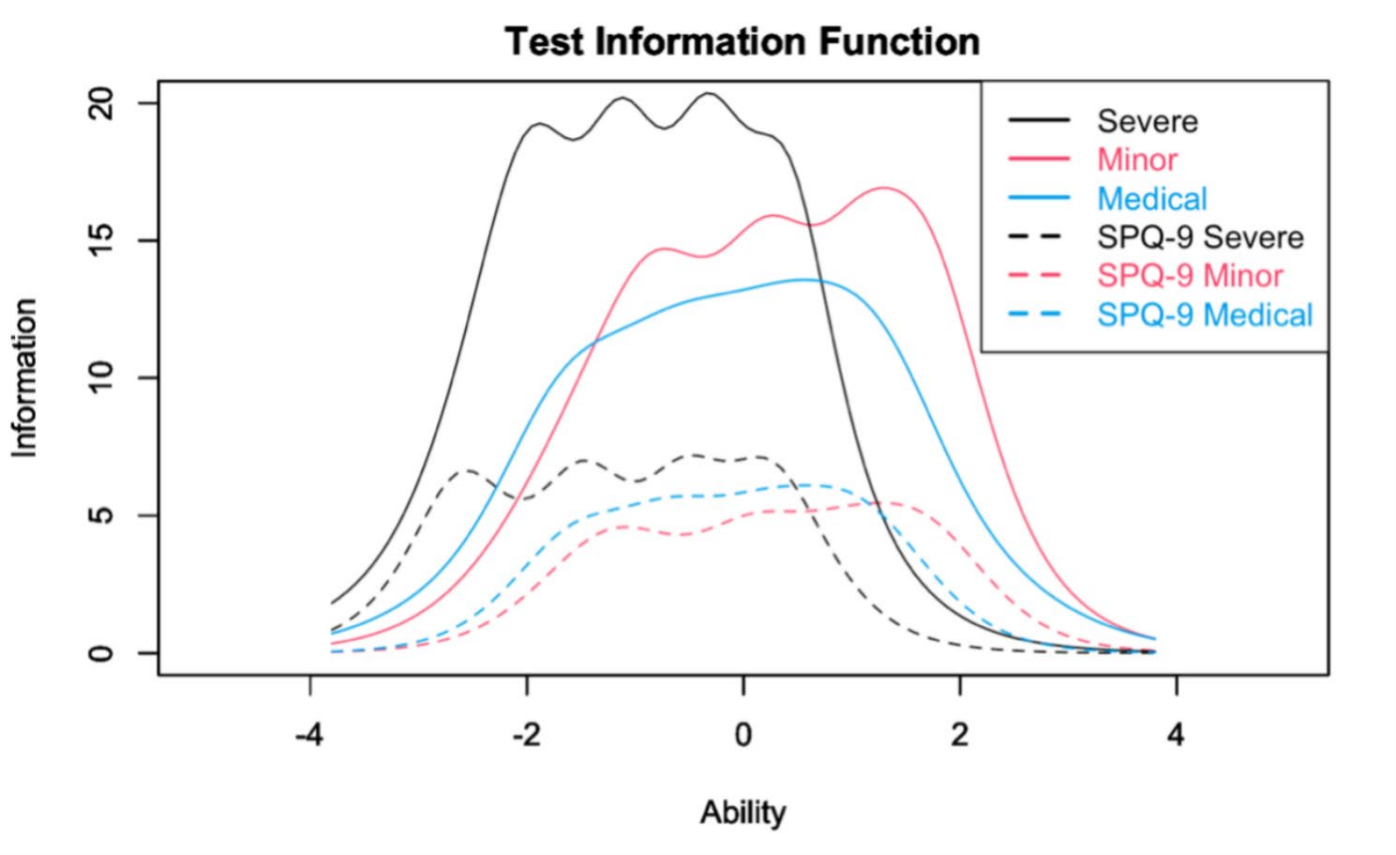
Comparison of FPQ-III and FPQ-9 by test information function for each subscale FPQ-III: fear of pain questionnaire III; FPQ-9: nine items of fear of pain questionnaire III

The test information functions for each subscale are shown in Fig. 3. Fear of severe pain has high accuracy in the negative characteristic value range. The shortened versions of each subscale were shown to be less informative but to maintain high accuracy in the same range of characteristic values as the original versions.

**Table 8:** Results of item analysis using unidimensional item response theory for each subscale

Items	Mean	S.D.	Polychoric correlation coefficient	Factor loading	beta.1	beta.2	beta.3	beta.4	discrimination (α)
I. Fear of Severe Pain									
1	4.20	1.04	0.78	0.69	-5.74	-3.40	-1.66	-0.06	2.33
3*	3.83	1.04	0.83	0.80	-6.88	-3.89	-0.74	1.77	3.34
5	3.54	1.14	0.75	0.74	-4.71	-1.74	-0.01	1.73	1.90
6	3.90	1.05	0.88	0.88	-8.04	-4.71	-1.41	1.76	4.32
9*	3.89	1.05	0.80	0.77	-5.48	-3.19	-0.82	1.24	2.49
10*	4.01	1.02	0.88	0.84	-7.56	-4.34	-1.46	1.00	3.49
13	4.47	0.86	0.79	0.54	-6.09	-4.58	-2.64	-0.92	2.44
18	3.76	1.14	0.79	0.79	-4.76	-2.26	-0.51	1.28	2.19
25	4.13	1.02	0.68	0.54	-5.41	-2.89	-1.09	0.14	1.46
27	3.61	1.12	0.69	0.64	-3.97	-1.97	-0.04	1.52	1.62
II. Fear of Minor Pain									
2	2.69	1.06	0.71	0.67	-2.70	-0.17	2.07	3.56	1.55
4	2.95	1.23	0.77	0.85	-2.67	-0.58	1.09	2.62	1.65
7	2.71	1.18	0.80	0.86	-2.62	-0.22	2.20	3.45	2.09
12	2.76	1.14	0.80	0.86	-3.34	-0.08	2.16	3.77	2.22
19*	2.62	1.17	0.83	0.92	-2.83	0.26	2.37	4.18	2.39
22	3.02	1.16	0.81	0.87	-3.95	-0.69	1.35	2.92	2.05
23*	2.85	1.17	0.80	0.89	-3.34	-0.44	1.86	3.61	2.28
24*	2.46	1.15	0.84	0.91	-2.74	0.64	3.56	5.15	2.99
28	2.39	1.19	0.86	0.97	-2.56	0.95	3.94	5.71	3.46
30	2.51	1.20	0.83	0.93	-2.29	0.26	2.90	4.56	2.61
III. Fear of Medical Pain									
8	2.07	1.19	0.76	0.73	-0.52	1.10	2.63	3.66	1.59
11	2.12	1.17	0.78	0.76	-0.77	1.12	2.54	3.96	1.72
14*	2.70	1.30	0.84	1.02	-2.59	-0.09	1.71	3.47	2.49
15	2.92	1.26	0.80	0.95	-3.20	-0.70	1.35	2.89	2.27
16	3.29	1.21	0.79	0.88	-3.97	-1.71	0.46	2.32	2.17
17*	3.26	1.24	0.83	0.96	-4.04	-1.75	0.57	2.42	2.52
20	3.78	1.10	0.74	0.70	-4.98	-2.59	-0.69	1.03	1.73
21*	3.09	1.18	0.83	0.94	-4.63	-1.33	1.22	3.09	2.63
26	3.30	1.22	0.76	0.83	-3.45	-1.64	0.34	2.11	1.86
29	2.84	1.25	0.80	0.91	-2.96	-0.42	1.40	2.93	2.11

* The items are included in FPQ-9

**Table 9:** Results of the item analysis using item response theory for the shortened version of the Fear of Pain Questionnaire

items	beta.1	beta.2	beta.3	beta.4	beta	Mean
I. Fear of Severe Pain						
3	-6.08	-3.41	-0.81	1.11	2.10	3.83
9	-7.03	-4.15	-1.27	1.15	2.87	3.89
10	-9.65	-5.45	-2.05	0.78	3.67	4.01
II. Fear of Minor Pain						
19	-3.56	-0.15	2.11	3.66	2.30	2.70
23	-3.11	0.21	2.39	4.22	2.59	3.26
24	-2.54	0.51	3.04	4.34	2.52	2.62
III. Fear of Medical Pain						
14	-2.54	-0.08	1.71	3.44	2.53	3.09
17	-4.36	-1.90	0.62	2.62	2.86	2.85
21	-4.35	-1.22	1.15	2.91	2.47	2.46

### Structural equation modeling (SEM)

The shortened 9-item version of the FPQ used for SEM was used, as the 9-item version also had the best CFA fit, and the test information curves showed that each subscale was highly accurate within the same range as the 30-item version.

Before performing SEM, the factor structure of the MDAS was checked: an EFA using SPSS showed that eigenvalues decay was 3.38, 0.49, and 0.37, supporting a one-factor structure according to the Guttman criterion and scree plot. The one-factor model was adopted because the Japanese version of the MDAS also showed a one-factor structure in previous studies [32, 33].

**Fig. 4:**
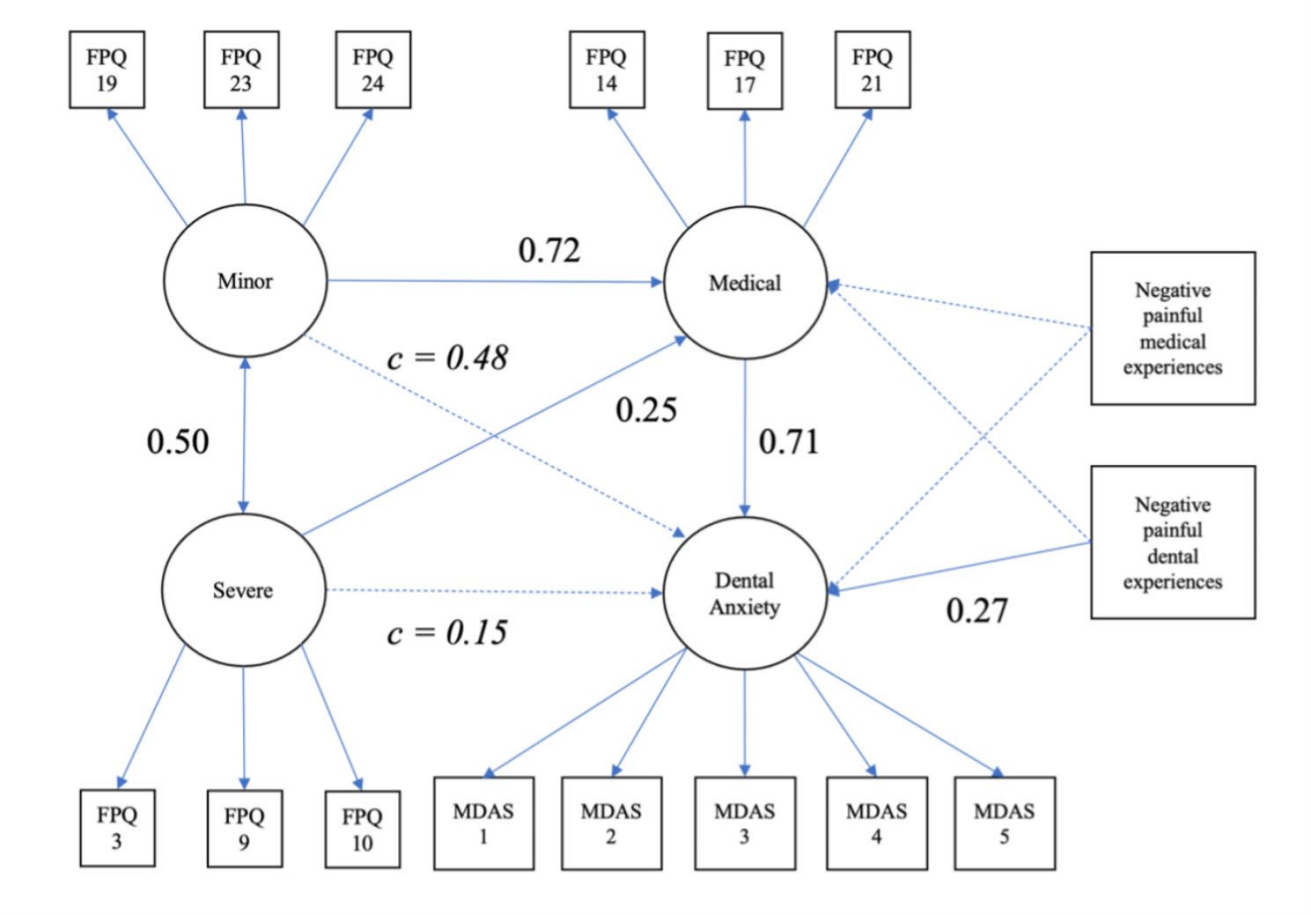
The estimated structural equation model with standardized coefficients for the interrelationships of dental anxiety, fear of severe/mild pain, and negative dental experiences. Dotted lines indicate non-significant paths. Path *c* indicates indirect effects

Figure 4 shows the results of the SEM for the hypothesized model. Both fears of severe and minor pain were shown to be indirectly related to dental anxiety via fear of medical pain. The magnitude of the indirect effect was higher for fears of minor pain than severe pain. Fears of medical pain were also unaffected by medical and dental pain experiences, while dental anxiety was only affected by painful dental experiences.

In addition, a multi-population analysis was conducted on the above model by the presence or absence of painful medical experiences and gender. The number of respondents who reported the absence of medical or dental experiences was n = 280 (Non-NegativeEx), while the number of respondents who reported the presence of medical or dental experiences was n = 120 (NegativeEx).

Table 10 shows the results of a multi-population analysis of the impact of painful dental experiences and gender on the model presented in Fig. 4.

**Fig. 5:**
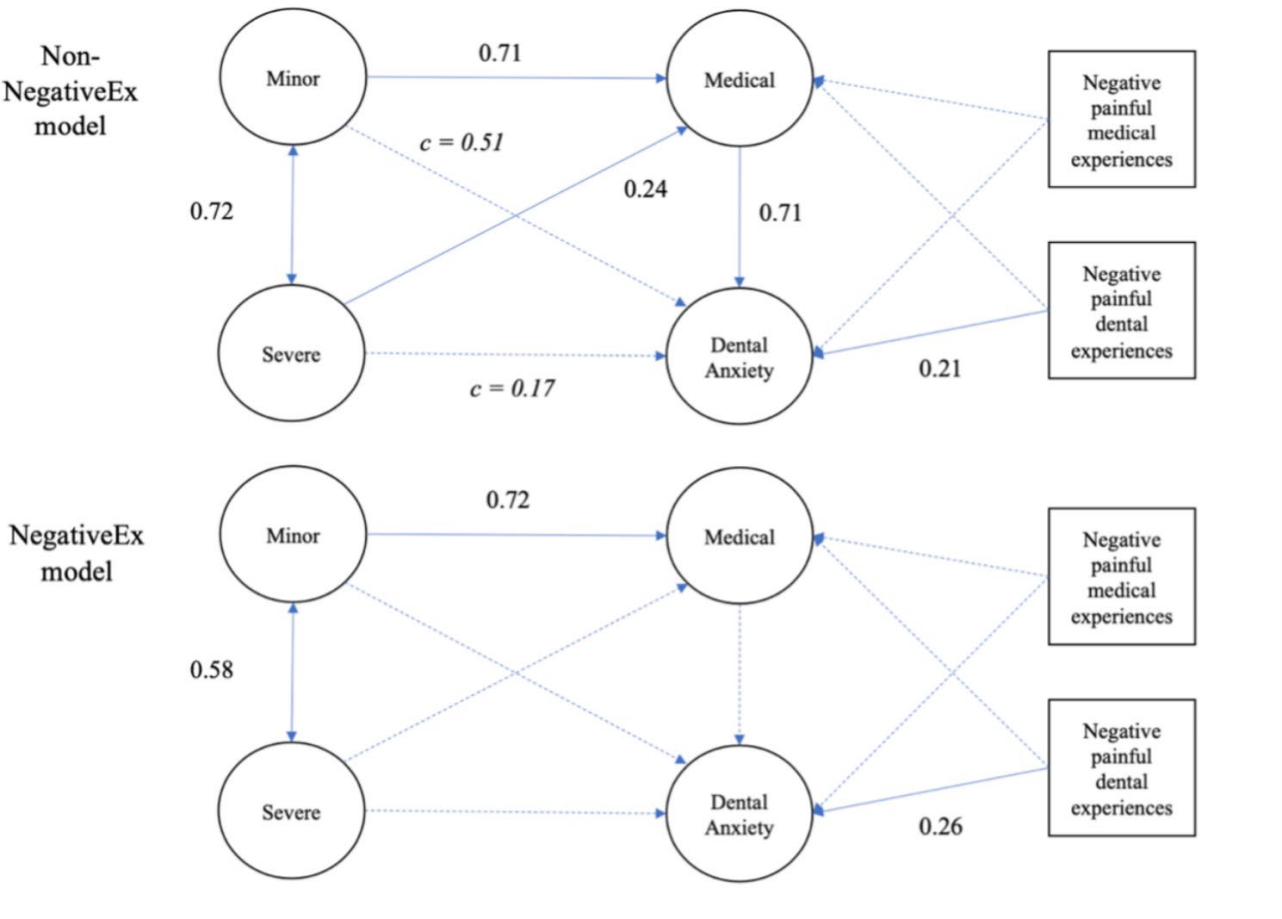
Comparison of the estimated structural equation model with standardized coefficients between with and without distressing medical/dental experiences. Dotted lines indicate non-significant paths. Path *c* indicates indirect effects

For negative dental or medical experiences, the configuration invariant model had a CFI 0.009 worse than the overall model, although slightly below the cut-off value; RMSEA, AIC, and BIC were worse than the overall model. Moreover, configural invariance was not selected. This means there is a difference in the path coefficient of the model between the two groups. The results of the individual models are shown in Fig. 5. In the group with no negative medical experience (Non-NegativeEx model), fear of minor and severe pain was associated with dental anxiety via fear of medical pain. In contrast, in the group with negative medical experiences, the three FPQ subscales were not significantly associated with dental anxiety (NegativeEx model).

**Fig. 6:**
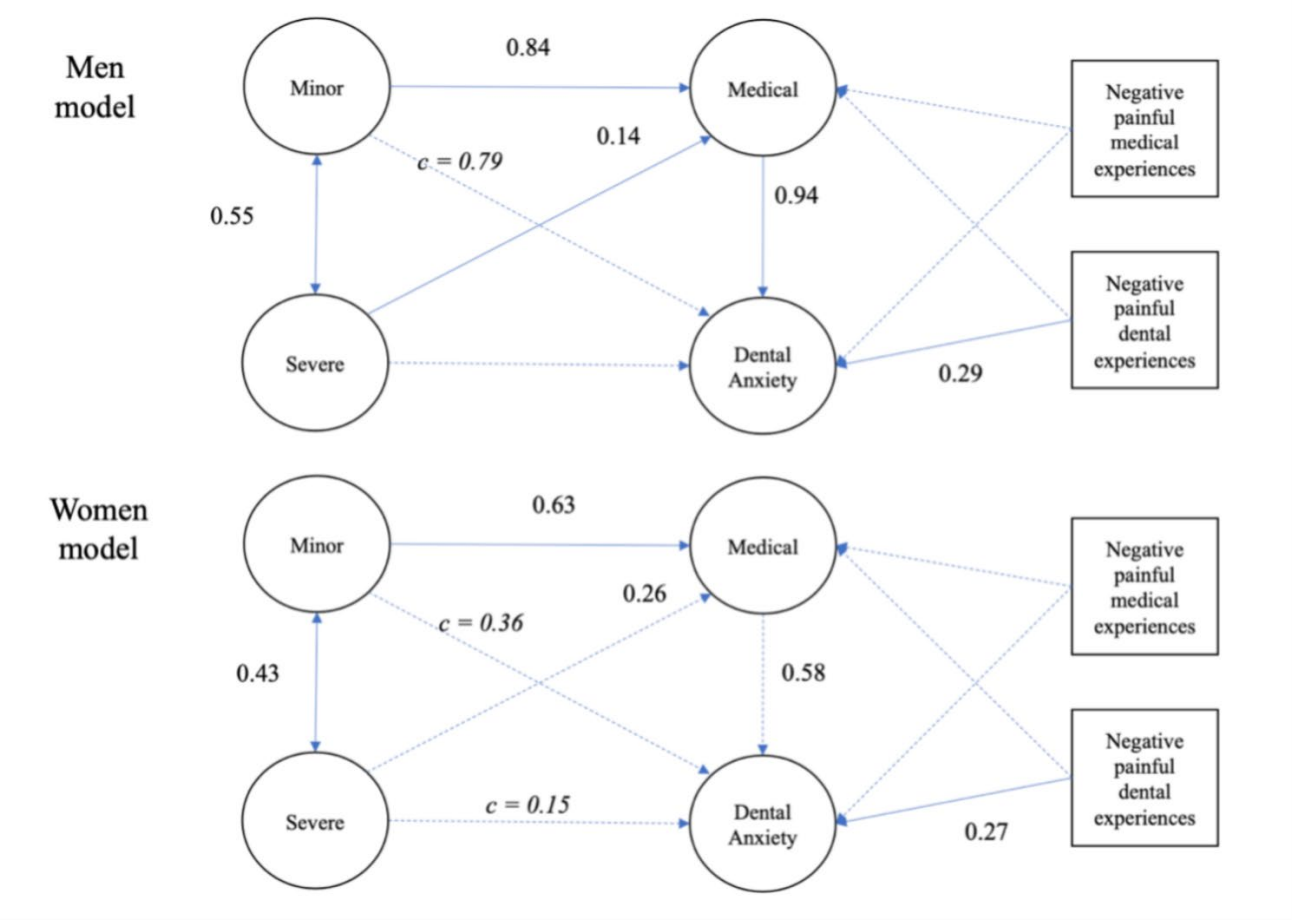
Comparison of the estimated structural equation model with standardized coefficients between gender. Dotted lines indicate non-significant paths. Path *c* indicates indirect effects

The configuration invariant model in gender also showed a 0.014 worse ΔCFI compared to the unconstrained model and worse RMSEA, AIC, and BIC, indicating a gender difference in structure. There was a significant relationship between fear of medical pain to dental anxiety for men and women. Men showed an indirect effect of fear of minor pain via fear of medical pain. For women, both fear of severe and minor pain showed significant indirect and overall effects via fear of medical pain (Fig. 6).

**Table 10:** Measurement and structural variance across the presence of negative dental or medical experiences and gender

Model	X^2^	df	CFI	RMSEA	SRMR	AIC	BIC
The presence of negative dental or medical experiences							
Overall model	292.6	95	0.943	0.072	0.055	14,251	14,459
NonEX model	283.9	95	0.922	0.084	0.051	9820	10,009
EX model	131.7	95	0.963	0.057	0.05	4448	4593
Configurel model	415.6	190	0.934	0.077	0.051	14,267	14,682
Metric model	430.8	209	0.935	0.073	0.057	14,244	14,584
Scalar model	456.5	219	0.93	0.074	0.059	14,250	14,549
Strict model							
Gender							
Overall model	292.6	95	0.943	0.072	0.055	14,251	14,459
Male model	228.6	95	0.926	0.084	0.053	6985	7156
Female model	209.3	95	0.933	0.078	0.069	7274	7445
Configurel model	437.9	190	0.929	0.081	0.061	14,259	14,674
Metric model	452.5	209	0.931	0.076	0.063	14,235	14,574
Scalar model	467.4	219	0.929	0.075	0.064	14,230	14,530
Strict model	496.2	233	0.925	0.075	0.064	14,231	14,474

Abbreviations: CFI, the comparative fit index; RMSEA, the root mean square error of approximation; SRMR, standardized root mean square residual; AIC, Akaike’s information criterion; BIC, Bayesian information criterion

## Discussion

The present study aimed to construct a Japanese version of the FPQ-III and examine its psychometric properties in a non-clinical Japanese sample. The present study aimed to construct a Japanese version of the FPQ-III and examine its psychometric properties in a non-clinical Japanese sample. The FPQ-III and the nine-item shortened version showed high retest reliability, internal consistency, and validity; the three-factor structure was better fitted by the nine-item shortened version compared to the original 30-item version, and the shortened version maintained high accuracy over as wide a range as the original version. Fear of minor pain suggested that even individuals without a negative medical/dental experience may be related to dental anxiety via fear of medical pain. Fear of severe pain tended to be higher in individuals with chronic pain and were also associated with dental anxiety via fear of medical pain in women.

The results of this study showed high internal consistency and retest reliability, as in other translated versions [6–9]. Of the three subscales, only fear of severe pain showed a low correlation with depression, indicating heterogeneity.

This is the first study to analyze FPQ-III items using IRT. The test information curves also show high accuracy for participants with a wide range of latent characteristics. These suggest that the shortened 9-item version may be helpful.

The results of the SEM showed that fears of minor pain were associated with dental anxiety via fears of medical pain. The model showed differences in structure depending on the presence or absence of a painful medical or dental experience. The group without a distressing medical experience also found that fear of severe pain and minor pain were associated with dental anxiety via fear of medical pain. This highlights the role of fear of pain as an individual characteristic in developing dental anxiety. In particular, it means that individuals who are particularly susceptible to fear of minor pain are also more likely to have medical pain, and dental anxiety even if they have not had a distressing medical experience. The results are consistent with the previous study fact that fears of minor pain have been shown to be associated with genetic loci [17]. The FPQ-9 version of fear of minor pain includes cutting fingers on paper, drinking hot drinks, gulping hot drinks, and getting irritating soap in both eyes, which are common experiences in daily life. The results of this study indicate that individuals who are anxious about these stimuli are associated with dental anxiety, regardless of their negative medical experience, and measuring this subscale may be able to predict which individuals are likely to develop dental anxiety.

Gender differences were found in the relationship between fear of pain and dental anxiety. While only fears of minor pain were associated with dental anxiety in men via fears of medical pain, women’s fears of severe pain were indirectly associated with dental anxiety. Fear of severe pain has been reported to be associated with patients with prolonged orofacial pain [16]. Most of the targets in the McNeil et al. report were women [13], these results suggest that fears of severe pain may be essential in treating dental anxiety and prolonged oral pain in women.

The factor structure of the nine-item version showed no difference between the groups with and without chronic pain. Only for severe pain-related fears the mean structure values were significantly higher in the group with chronic pain than those without. This is similar to previous studies comparing patients with orofacial pain with healthy controls [13]. Fear of pain may play a role in avoiding danger but can lead to a transition from acute to chronic pain and pain-related life problems. Addressing fears of severe pain may be a point of intervention for patients with chronic pain.

The present study has several limitations. The first is the low response rate due to the nature of web-based surveys. This study was conducted via the Internet on monitors registered with a private research company. The characteristics of such web-based surveys are that the number of valid responses can be easily obtained. However, the response rate is low, and the target participants are limited to those with access to the Internet, reducing the sample’s representativeness [26]. Measures are sometimes taken to reduce this disadvantage by using the quota method, which allocates the target participants into specified proportions by such as age, gender, and place of residence. This study also adopted measures to increase the generalizability of the results by equally allocating age and gender, although the recovery rate was low at 4.4%. Second, the participants in this study were from the general population, and the structure may differ, for example, in patients seen in pain clinics or those with severe dental anxiety. Third, as participants voluntarily participate in the study, there may be a volunteer bias, whereby they are more confident of their health than the general population. Fourth, the present study is observational, so a causal relationship cannot be determined. In addition, responses to questions about experience, e.g., negative dental treatment, may obtain recall bias. However, the study has strengths in terms of sufficient sample size, a wide age range, an equitable gender ratio, and the use of IRT.

## Conclusion

In summary, the Japanese version of the FPQ-III showed that the shortened nine-item version is particularly useful and highly accurate for targets with a wide range of characteristic values. Furthermore, it was suggested that fear of severe pain may be an important intervention point for those with chronic pain, and fear of minor pain for those with dental anxiety, regardless of gender or painful medical or dental experiences.

## Data Availability

The datasets used and/or analysed during the current study are available from the corresponding author on reasonable request.

## Abbreviations

FPQ-III: Fear of Pain Questionnaire-III
IRT: Item Response Theory
ICC: Intraclass Correlation Coefficient
MDAS: Modified Dental Anxiety Scale
CFI: Comparative Fit Index
RMSEA: Root Mean Square Error of Approximation
SRMR: Standardized Root Mean Square Residual
AIC: Akaike Information Criterion
BIC: Bayesian Information Criterion
